# Development of the Autofluorescence Endoscope Imaging System

**DOI:** 10.1155/DTE.5.65

**Published:** 1999

**Authors:** Rensuke Adachi, Tetsuya Utsui, Koichi Furusawa

**Affiliations:** 1 Research and Designing Department Medical Instrument Division Asahi Optical Co. Ltd. 2-36-9 Maenocho Itabashi-ku Tokyo 174 Japan; 2 Research and Development Division Asahi Optical Co. Ltd. 2-36-9 Maenocho Itabashi-ku Tokyo 174 Japan

## Abstract

It is a well known fact that light emitted at a specific wavelength induces fluorescence in the human body. This kind of fluorescence is called autofluorescence. The application of autofluorescence diagnosis, on the other hand, is a more complicated system designed to detect faint autofluorescence inherent in tissues/cells. We have adopted this autofluorescence diagnosis method and developed a new autofluorescence endoscope imaging system called the SAFE-1000. Normal mucosa emitting autofluorescence appears green on the monitor, while abnormal mucosa shows a dark image caused by the lack of autofluorescence.


*Diagnostic and Therapeutic Endoscopy*, Vol. 5, pp. 65–70
Reprints available directly from the publisher
Photocopying permitted by license only

© 1999 OPA (Overseas Publishers Association) N.V.
Published by license under
the Harwood Academic Publishers imprint,
part of The Gordon and Breach Publishing Group.
Printed in Singapore.

### Development of the Autofluorescence Endoscope Imaging System

RENSUKE ADACHI[a,*], TETSUYA UTSUI[a] and KOICHI FURUSAWA[b]

[a]*Research and Designing Department, Medical Instrument Division;* [b]*Research and Development Division, Asahi Optical Co., Ltd. 2-36-9 Maenocho Itabashi-ku,.Tokyo 174, Japan*

**It is a well known fact that light emitted at a specific wavelength induces fluorescence in the human body. This kind of fluorescence is called autofluorescence. The application of autofluorescence diagnosis, on the other hand, is a more complicated system designed to detect faint autofluorescence inherent in tissues/cells. We have adopted this autofluorescence diagnosis method and developed a new autofluorescence endoscope imaging system called the SAFE-1000. Normal mucosa emitting autofluorescence appears green on the monitor, while abnormal mucosa shows a dark image caused by the lack of autofluorescence.**

*Keywords:* Autofluorescence, Bronchoscope, Carcinoma *in situ*, Image intensifier

### 1. INTRODUCTION

Endoscopic application of fluorescence for medical detection of carcinoma has recently attracted considerable attention. These spectroscopic techniques can be classified into two basic types. One method is called photodynamic diagnosis (PDD) [1,2,3] using chemicals called photosensitizers that react to various wavelengths of light. The other method is autofluorescence diagnosis, hereafter referred to as AFD, employing inherent tissue/mucosal autofluorescence without the use of additional photosensitizers.

PDD using tumor-affinitive photosensitizers is the easier of the two techniques to apply. However, after intravenous administration of these chemical photosensitizers, a patient must remain in a darkened environment until these light sensitive chemicals have metabolized to a safe level. Actual diagnosis via this method is somewhat more difficult due to the fact that fluorescence from the photosensitizers interferes with the autofluorescence from normal mucosa.

The application of AFD, on the other hand, is a more complicated system designed to detect faint autofluorescence inherent in tissues/cells. Since it does not require the use of any photosensitizers, it produces fewer side effects. Furthermore, it can be performed in conjunction with conventional endoscopy. With safety concerns being the primary

* Corresponding author.

factor, Asahi Optical Co., Ltd. has adopted this AFD method and developed a new autofluorescence endoscope imaging system called the SAFE-1000.

It is a well known fact that light emitted at a specific wavelength induces fluorescence in the human body [3–9]. This kind of fluorescence is called autofluorescence. Typically, a greater amount of autofluorescene occurs with normal mucosa and little fluorescene is exhibited by abnormal tissue. Applying this same principle, Pentax has developed a new autofluorescence endoscope imaging system (Pentax SAFE-1000) without the application of any photosensitizing agents or use of any laser.

By utilizing the same white light used in conventional endoscopy through a pass filter that allows passage of only specific wavelengths, the SAFE-1000 collects blue light and delivers it endoscopically to mucosa to excite tissue autofluorescence. By detecting and amplifying the faint autofluorescence at a green wavelength from normal mucosa, a highly sensitive video camera incorporated into the SAFE-1000 can show the intensified green autofluorescent image on a color monitor. However, for abnormal tissue, the autofluorescence at the green wavelength is significantly diminished, making it appear dark on the video monitor. It can then be easily distinguished from the more intense autofluorescence obtained from normal mucosa [2].

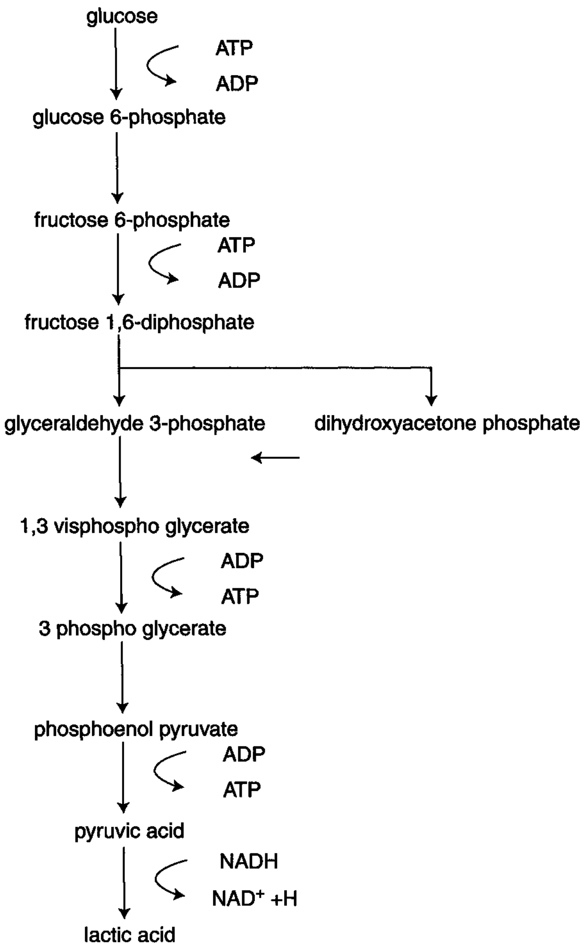

FIGURE 1 The overproduction of lactic acid through glycolysis.

### 2. EXPERIMENTAL MODEL

Otto Warburg was a German biochemist who discovered that there are considerable biochemical differences between normal cells and cancer cells [10]. For instance, he found that cancer cells produce more lactic acid than normal cells. This phenomenon was confirmed both *in vivo* and *in vitro* studies. Several investigations have been performed on the overproduction by cancerous cell of lactic acid, called the Warburg effect. Though the entire process has not been completely defined, it has been confirmed that this overproduction (of lactic acid) is generated by glucose through a glycolysis pathway (Fig. 1).

This increase in the overproduction of lactic acid through glycolysis is due not so much to decreased enzymatic action but to an increase in fermenting activity. The fact that tumors consume more glucose than is usually required for normal growth indicates that there is some malfunction in the mechanism at which the cells control the speed of absorption of glucose. It has actually been confirmed that the cellular absorption speed of glucose is increased in abnormal cells.

The following common substances found in the human body are known to exhibit tissue autofluorescence (Table I).

TABLE I Fluorescent substances in the human body

| Fluorescent substance | Exitation light wavelength (nm) | Fluorescent wavelength (nm) |
|---|---|---|
| Tryptophan | 280 | 340 |
| Collagen | 325 | 380 |
| Elastin | 410 | 440 |
| NADH | 365 | 470 |
| Flavin | 440 | 520 |
| Porphyrin | 400 | 630, 690 |

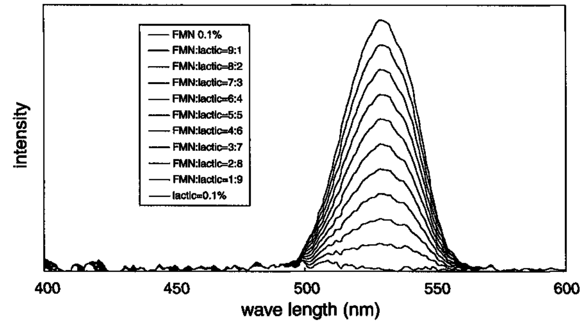

FIGURE 2 Fluorescence spectrum by changing the ratios of FMN and lactic acid.

The substances that participate in the AFD are not specified. In our study, we noted that when the fluorescent substance is mixed with other substances, the peak wavelength of the fluorescence is not changed and is merely attenuated.

In this study, flavin and its derivative, flavin mono nucleotide (FMN), have been selected because flavin is associated with cellular/biochemical metabolism and fluoresces in the green wavelength. Based upon the Warburg effect, it is believed that the different metabolic processes between normal mucosa and tumors might have some effects on the spectral emission of their autofluorescene. We have therefore developed the following experimental model (Fig. 2).

Figure 2 shows the fluorescence intensity exhibited by several mixtures of FMN and lactic acid due simply to changing their proportions. At a molecular level, FMN emits autofluorescence while lactic acid does not. Depending upon the ratios in each mixture, the fluorescence intensity of each mixture varies. Flavin and its derivatives such as FMN or FAD are enzymes or coenzymes that are widely distributed throughout the human body and are strongly associated with oxidation–reduction reactions. Consequently, it is the molecular make-up and their different metabolic processes which produce the different relative densities of autofluorescent and non-autofluorescent substances. This seems to be one of the factors that would then contribute to the different intensities in autofluorescence inherent in various tissues. Moreover, it is a well-known fact that blood flow increases in tumors and cancerous tissues. Blood can be considered as a non-autofluorescent substance in this case, because it does not emit any autofluorescence at the observed wavelength. Therefore, the increased blood flow in tumors seems to be one of the primary factors that could cause different intensities of autofluorescence in various types of tissues. As a result, it would appear that the SAFE-1000 could aid in the detection of tumors by identifying metabolic differences, due to changes in cellular abnormalities, via autofluorescence.

### 3. PENTAX SAFE 1000 (A NEW AUTOFLUORESCENCE ENDOSCOPE IMAGING SYSTEM)

### 3.1. System Configuration

(1) Target Area: bronchial tubes.
(2) Equipment (Fig. 3):
  (1) A bronchofiberscope (Pentax FB-18RX).
  (2) An excitation light source (Pentax LX-750AF) incorporating a 75 W xenon lamp and an excitation filter (EX filter). The EX filter is automatically inserted to or removed from the light path with the changeover switch on the camera box. Through the EX filter, the light source delivers excitation blue light for antofluorescence endoscopy. Without the filter, the light source delivers white light for conventional endoscopy.

(3) A video camera incorporating
- an endoscopic TV camera with a 410000-pixel 1/2″ CCD;
- a fluorescence TV camera;
- light path changing optics (prism);
- a fluorescence filter (FL filter);
- an image intensifier which amplifies faint fluorescence signal thousands of times;
- a changeover witch on the TV camera box. The switch changes the camera for endoscopic or fluorescence use and simultaneously inserts or removes the EX filter to or from the light path;
- an image intensifier control switch (AUTO FL CONTROL) on the TV camera box. The switch can adjust the brightness of fluorescence images.

(4) An image intensifier controller (Pentax SAFE-1000c) which
- processes fluorescence images (frame average addition of two-frame, four-frame, eight-frame or live image with the recursive filter);
- changes the monochrome fluorescence signal to the G signal;
- adjusts the brightness and contrast of the fluorescence images.

(5) Peripherals
- a monitor;
- a video cassette recorder;
- a video printer.

### 3.2. Operation

#### *(1) Conventional Endoscopy*

By setting the changeover switch on the camera box to "NORMAL", conventional endoscopic procedure can be performed. The 75 W xenon lamp in the

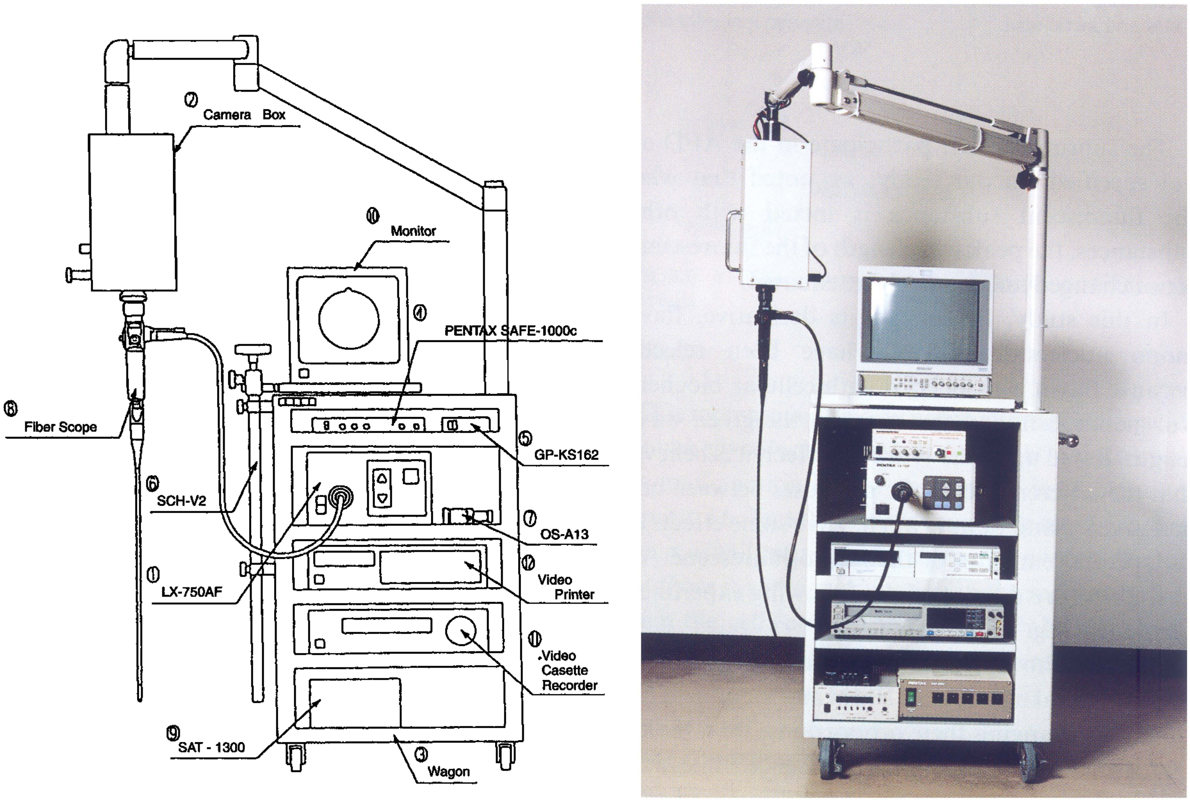

FIGURE 3 Pentax SAFE-1000.

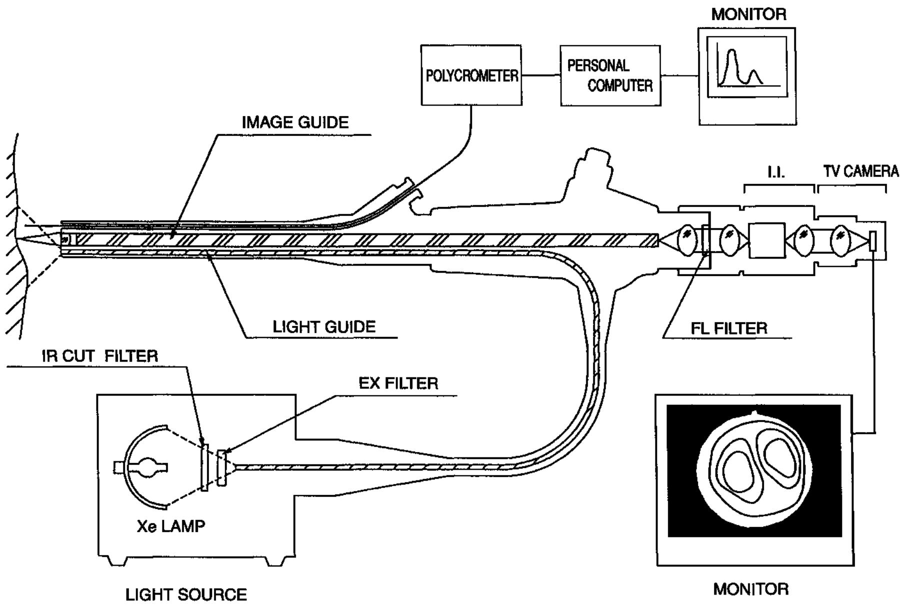

FIGURE 4 The optical system of endoscopic autofluorescence imaging.

light source emits white light. Infrared light is eliminated by the infrared (IR) cut filter. Bypassing the EX filter, white light is transmitted via a light guide to the target area. Images obtained with an objective lens are transmitted via a fiberoptic image guide back to the eyepiece of the endoscope and guided through the prism to endoscopic TV camera.

### *(2) Autofluorescence Endoscopy*

By setting the changeover switch on the camera box to "AUTO FL", an autofluorescence endoscopic procedure can be performed (Fig. 4). The white light from the 75 W xenon lamp is passed by the IR cut filter and then by the EX filter which specifically passes 420–450 nm excitation light. The excitation light is then transmitted via a light guide to the target area.

Images obtained with an objective lens are transmitted via a fiberoptic image guide back to the eyepiece of the endoscope and guided through the prism to the FL filter which specifically passes 490–590 nm fluorescence signal. The selected signal is then amplified by the image intensifier thousands of times, and by means of a video camera, appears as fluorescent images on the monitor. Normal mucosa emitting autofluorescence appears green on the monitor, while abnormal mucosa shows a dark image caused by the lack of autofluorescence, which allows for easier recognition of carcinoma in situ [11].

### *(3) Features*

- Fluorescence observation without using any photosensitizers;
- Detection of early cancers not superficially visible;
- Safer and less expensive without using lasers;
- Possible to use conventional endoscopes;
- A compact and space-saving system;
- Easy to changeover between conventional and fluorescent images, which enables moreaccurate diagnosis.

### 4. CONCLUSION

The autofluorescence endoscope imaging system, Pentax SAFE-1000, should aid physicians in the ability to recognize areas of early cancer and squamous metaplasia and in the detection of precancerous lesions which cannot be identified by conventional endoscopy. Compared to other fluorescence techniques requiring photosensitizers, this method is more appealing to and less traumatic for patients.

### Acknowledgements

The authors thank Dr. Harubumi Kato, M.D. Department of Surgery, Tokyo Medical College for his keen insight and invaluable advice in the clinical application of this system.

### References

[1] Lam, S., MacAulay, C., LeRiche, J., Ikeda, N. and Palcic, B. Fluorescence image of early lung cancer. *SPIE* 1994; **2324**(3): 2–8.

[2] Kim, K., Ikeda, N., Okunaka, T., Furukawa, K., Konaka, C. and Kato, H. Autofluorescence diagnosys of precancer/early cancer of the bronchus. *Japan Society for Laser Medicine* 1996; **17**(1): 81–85.

[3] Kato, H., Konaka, C., Okunaka, T., Furukawa, K. and Ikeda, N. Early diagnosis of central type lung cancer with bronchoscope. *Medicina Thoracalis* 1997; **50**: 131–143.

[4] Alfano, R.R., Pradhan, A. and Tang, G.G. Optical spectroscopic diagnosis of cancer and normal breast tossues. *J. Opt. Soc. Am. B* 1989; **6**(5): 1015–1023.

[5] Hung, J., Lam, S., LeRiche, J.C. and Palcic, B. Autofluorescence of normal and malignant bronchial tissue. *Laser in Surgery and Medicine* 1991; **11**: 99–105.

[6] Alfano, R.R., Tang, G.G., Pradhan, A., Lam, W., Choy, D.S.J. and Opher, E. Fluorescence spectra from cancerous and normal human and lung tissues. *IEEE Journal of Quantum Electronics* 1987; **QE-23**(10): 1806–1811.

[7] Alfano, R.R., Tata, D.B., Cordero, J., Tomashefsky, P., Longo, F.W. and Alfano, M.A. Laser induced fluorescence spectroscopy from native cancerous and normal tissue. *IEEE Journal of Quantum Electronics* 1984; **QE-20**(12): 1507–1511.

[8] Lam, S., Hung, J., MacAulay, C., Jaggi, B. and Palcic, B. Fluorescence image of early lung cancer. *Annual International Conference of the IEEE Engineering in Medicine and Biology Society* 1990; **12**(3): 1142–1143.

[9] Jacques, S.L. Tissue fluorescence. *SPIE* 1995; **2371**(269): 2–13.

[10] Watson, Hopkins, Roberts, Steitz, and Weiner *Molecular Biology of the Gene*. California: The Benjamin/Cummings Publishing, 1987.

[11] Kato, H., Okunaka, T., Ikeda, N. and Konaka, C. Application of simple imaging technique for fluorescence bronchoscope: preliminary report. *Diagnostic and Therapeutic Endoscopy* 1994; **1**: 79–81.

